# CaMK II γ down regulation protects dorsal root ganglion neurons from ropivacaine hydrochloride neurotoxicity

**DOI:** 10.1038/s41598-017-05678-2

**Published:** 2017-07-12

**Authors:** Xian-Jie Wen, Xiao-hong Li, Heng Li, Hua Liang, Chen-Xiang Yang, Han-Bing Wang

**Affiliations:** 1 Department of Anesthesiology, The First People’s Hospital of Foshan & Foshan Hospital of Sun Yat-sen University, Foshan, 528000 Guangdong Province China; 2 Department of Anesthesiology, The sixth affiliated hospital of Guangzhou medical university, Qinyuan, 511518 Guangdong Province China

## Abstract

T-type calcium channels are intimately involved in the local anesthetics neurotoxicity. Does CaMKIIγ regulate T-type calcium currents in local anesthetics neurotoxicity? This study generated pAd-CaMKIIγ and pAd-shRNA adenovirus vectors to up- and down-regulate CaMKIIγ mRNA expression in dorsal root ganglion neurons (DRG). Normal DRG (Normal group), empty vector DRG (Empty vector group), pAd-CaMKIIγ DRG (pAd-CaMKIIγ group) and pAd-shRNA DRG (pAd-shRNA group) were treated or untreated with 3 mM ropivacaine hydrochloride for 4 h. Cell viability, apoptosis rate, CaMKIIγ, pCaMKIIγ, Cav3.2, and Cav3.3 expression were detected. Ultrastructural changes in DRG were observed under a transmission electron microscope. The results demonstrated that the cell viability of DRG treated with ropivacaine hydrochloride decreased markedly, the apoptosis rate, CaMKIIγ, pCaMKIIγ, Cav3.2, Cav3.3 expression increased significantly. CaMKIIγ up-regulation aggravated ropivacaine hydrochloride-induced cell damage and increased Cav3.2 and Cav3.3 expression. In conclusion, CaMKIIγ regulated Cav3.2 and Cav3.3 expression in DRG, which was involved with ropivacaine hydrochloride-induced cell injury.

## Introduction

Ropivacaine hydrochloride is a widely used local anesthetic in clinical anesthesia and pain management because of good separation of the sensory and motor nerve block, few systemic reactions and lower cardiac toxicity^1–4^. However, high concentrations or long exposure times of local anesthetics to neurons also results in neuronal damage^5, 6^. Ropivacaine hydrochloride is commonly used in peripheral nerve block or spinal analgesia with a long exposure time, but it also causes nerve damage and abnormal sensations similar to other local anesthetics^7, 8^. A multicenter study reported that the occurrence rate of local anesthetic-induced transient neurological syndrome (TNS) was 8.1%^9^. TNS is reversible, but it causes patient discomfort with spastic and radioactive burning pain.

The precise mechanism of local anesthetic neurotoxicity is not clear; it generally involves the physicochemical properties of the anesthetic, intracellular calcium concentration, cell apoptosis, inflammation and neurotrophic factors^10–13^. Intracellular calcium overload is an important factor in local anesthetic neurotoxicity. Gold *et al*. found that local anesthetics increased calcium ion concentrations of DRG neurons, which resulted in nerve injury^14^. Extracellular calcium chelating agents improved local anesthetic-induced nerve injury^14^. These data demonstrate that calcium influx is closely related to local anesthetic injury.

Calcium/calmodulin-dependent protein kinase II (CaMKII) is a multifunctional protease with multiple phosphorylation sites. CaMKII is the primary component of the postsynaptic density (PSD), and it is widely distributed in muscle, nerve and immune tissue^15–17^. CaMKII is divided into four subtypes: α, β, γ, and δ. CaMKII phosphorylation is associated with numerous physiological and pathological processes^18–20^. Our previous study demonstrated that ropivacaine hydrochloride induced the up-regulation of mRNA expression of all four CaMK II subtypes in rat dorsal root ganglion (DRG) neurons^21^. We also found that low-voltage-dependent calcium channels, namely, T-type calcium channels, were intimately involved with local anesthetic neurotoxicity^22, 23^. T-type calcium channels act as pacemakers to regulate intracellular calcium ion levels, which control CaMKII activity^17^. Previous studies demonstrated a close relationship between T-type calcium channels and CaMKIIγ, in which an up-regulation of CaMKIIγ increased T-calcium currents, and vice versa^24, 25^. T-type calcium channels are intimately involved in the local anesthetics neurotoxicity. Does CaMKIIγ regulate T-type calcium currents in local anesthetics neurotoxicity?

This study examined DRG injury induced by different ropivacaine hydrochloride concentrations and exposure times and investigated the role of CaMK II γ in ropivacaine hydrochloride-induced DRG injury. Our results demonstrated that the up-regulation of CaMK IIγ in DRG increased ropivacaine hydrochloride neurotoxicity and vice versa.

## Results

### Cell viability of ropivacaine hydrochloride-treated cells

**Figure 1:**
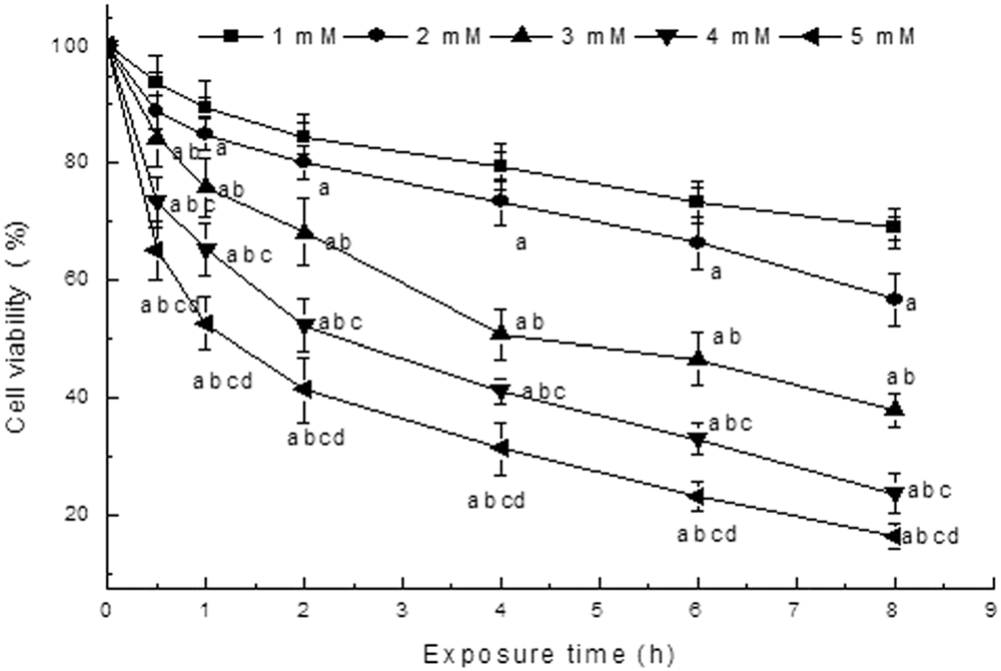
The viability of DRG cells after treatment with different ropivacaine hydrochloride concentrations and exposure times ($\overline{{\rm{x}}}$ ± s, %, n = 8). ^*a*^ *P* < 0.05 vs. 1 mM group, ^*b*^ *P* < 0.05 vs. 2 mM group, ^*c*^ *P* < 0.05 vs. 3 mM group, ^*d*^ *P* < 0.05 vs. 4 mM group.

To investigate the effects of ropivacaine hydrochloride on the DRG cell viability, We measured the viability of DRG cells treated with different ropivacaine hydrochloride concentrations and exposure times using the MTT method. Figure 1 shows the results. Increased ropivacaine hydrochloride concentrations and exposure times decreased cell viability. DRG cells treated with 3 mM ropivacaine hydrochloride for 4 h reduced cell viability to 50%. Cell viability decreased sharply at high concentrations or longer treatment times. Therefore, this study used the 3 mM ropivacaine hydrochloride and a 4-h exposure time in the subsequent experiments.

**Figure 2:**
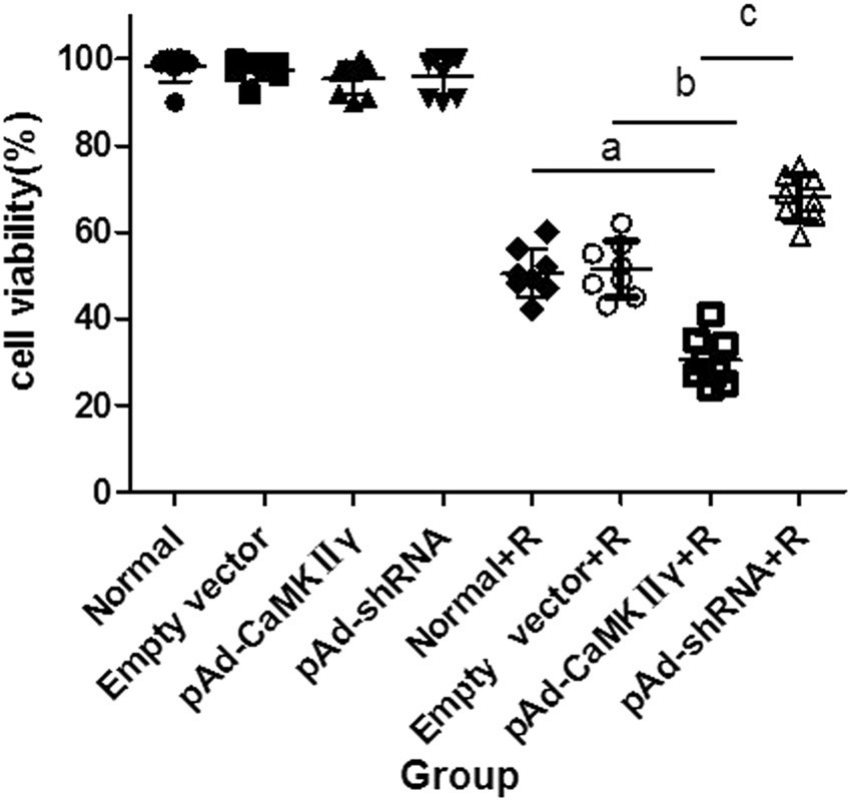
The effects of ropivacaine hydrochloride on the cells in each group. ($\overline{{\rm{x}}}$ ± s, %, n = 8). ^*a*^ *P* < 0.05 vs. Normal + R group, ^*b*^ *P* < 0.05 vs. Empty vector group, ^*c*^ *P* < 0.05 vs. pAd-CaMK IIγ group.

Cells in the Normal group, Empty vector group, pAd-CaMK IIγ group and pAd-shRNA group were treated with 3 mM ropivacaine hydrochloride for 4 h to examine the effects of CaMK IIγ on nerve cell injury. The viability of DRG cells decreased in each group treated with ropivacaine hydrochloride (Fig. 2). However, cell viability in the pAd-CaMK IIγ + R group was lower than the Normal + R and Empty vector + R groups. Notably, cell viability in the pAd-shRNA + R group was higher than the Normal + R and Empty vector + R groups. These data indicate that the up-regulation of CaMK IIγ expression resulted in neuronal injury.

### Cell apoptosis rate

**Figure 3:**
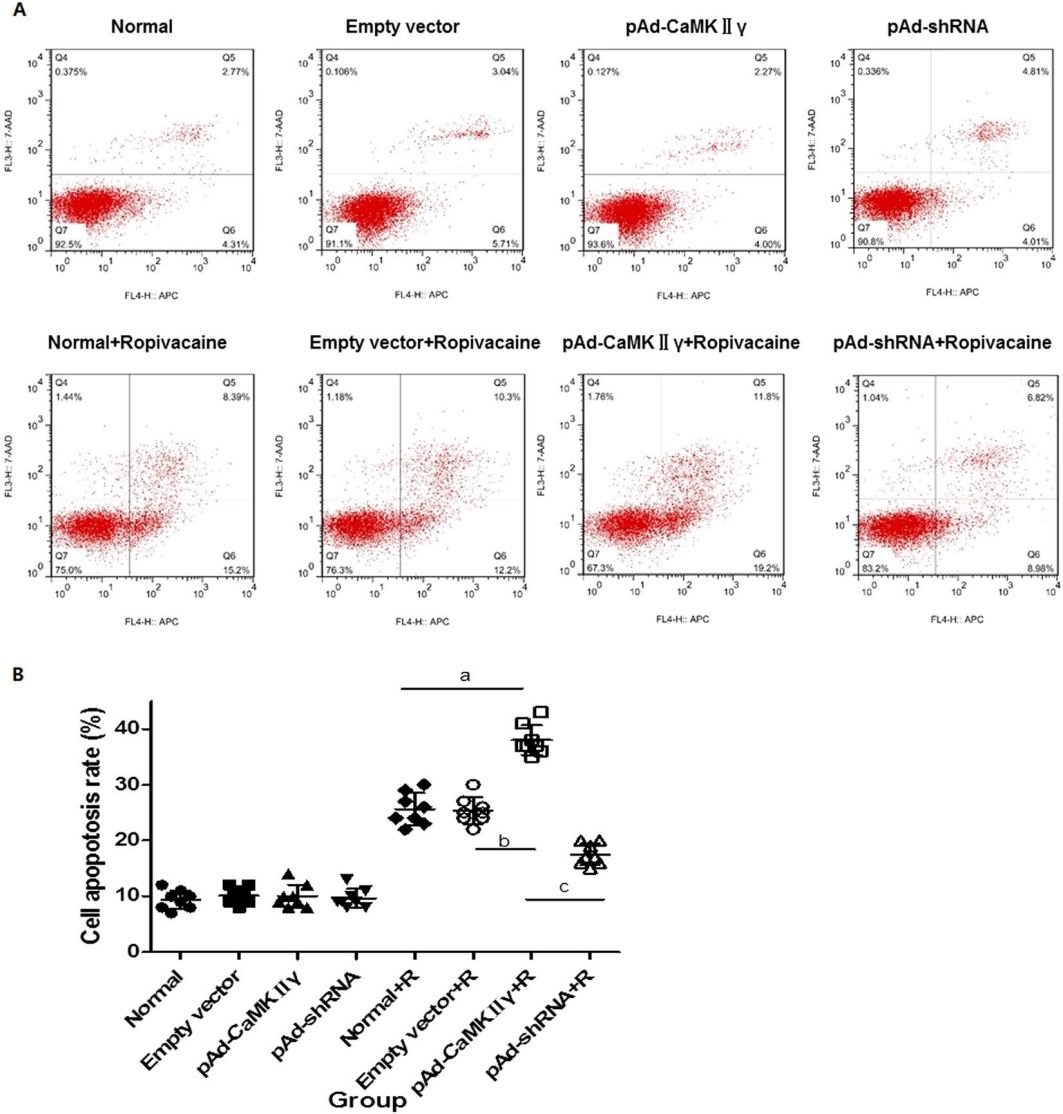
The apoptosis rate of the cells in every group. (**A**) Representative results of the cell apoptosis rate in every group detected by flow cytometry. (**B**) The statistic data of the cell apoptosis rate ($\overline{{\rm{x}}}$ ± s, %, n = 8). ^*a*^ *P* < 0.05 vs. Normal + R group, ^*b*^ *P* < 0.05 vs. Empty vector group, ^*c*^ *P* < 0.05 vs. pAd-CaMKIIγ group.

Cells treated with ropivacaine hydrochloride increased the apoptosis rate compared to the untreated group. The apoptosis rate in the pAd-CaMKIIγ + R group increased significantly compared to the Normal + R and Empty vector + R groups. However, the apoptosis rate of cells in the pAd-shRNA + R group was lower than the Normal + R and Empty vector + R groups because of the down-regulation of CaMKIIγ. These data suggest that the up-regulation of CaMKIIγ expression aggravated ropivacaine hydrochloride-induced damage to DRG cells, and CaMKIIγ down-regulation protected DRG cells (Fig. 3).

### Ultrastructural changes

**Figure 4:**
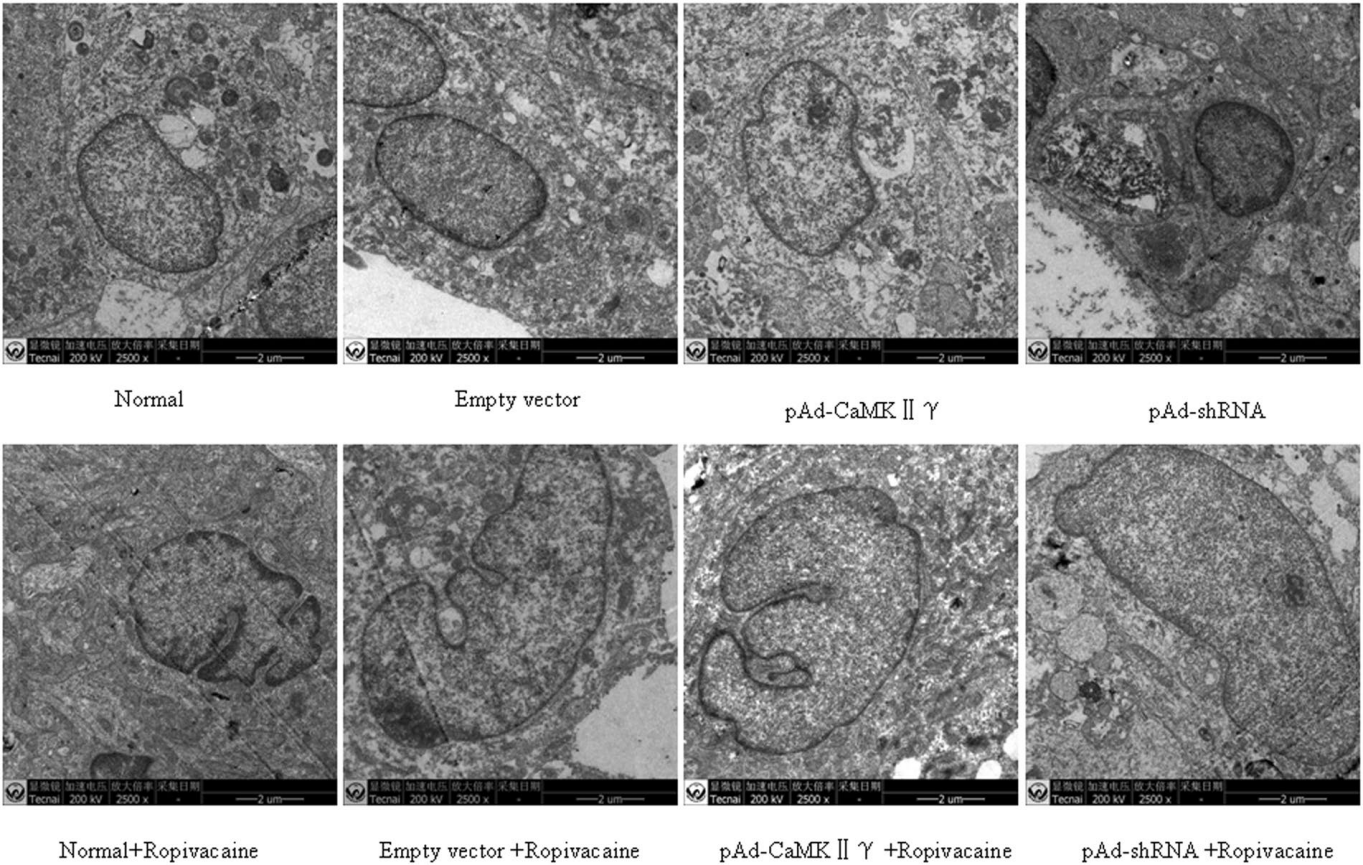
Ultrastructural changes in cells in each group using transmission electron microscopy (200w, ×2500).

Ropivacaine hydrochloride induced DRG cell injury and decreased cell viability, which resulted in apoptosis and cell death. Ropivacaine hydrochloride also caused ultrastructural changes. Organelle structure in untreated groups was complete, and mitochondria exhibited a regular shape and an intact envelope with no swelling (Fig. 4). Ropivacaine hydrochloride treatment induced mitochondrial swelling, the loss of mitochondrial crista, membrane rupture and atrophy. Organelle structure was incomplete and degraded by lysosomal enzymes. The ultrastructural damage of cells in the pAd-CaMKIIγ + R group was worse than the Normal + R and Empty vector + R groups. However, this damage was lower in the pAd-shRNA + R group than the Normal + R and Empty vector + R groups (Fig. 4).

### CaMKIIγ, Cav3.2, and Cav3.3 mRNA expression

**Figure 5:**
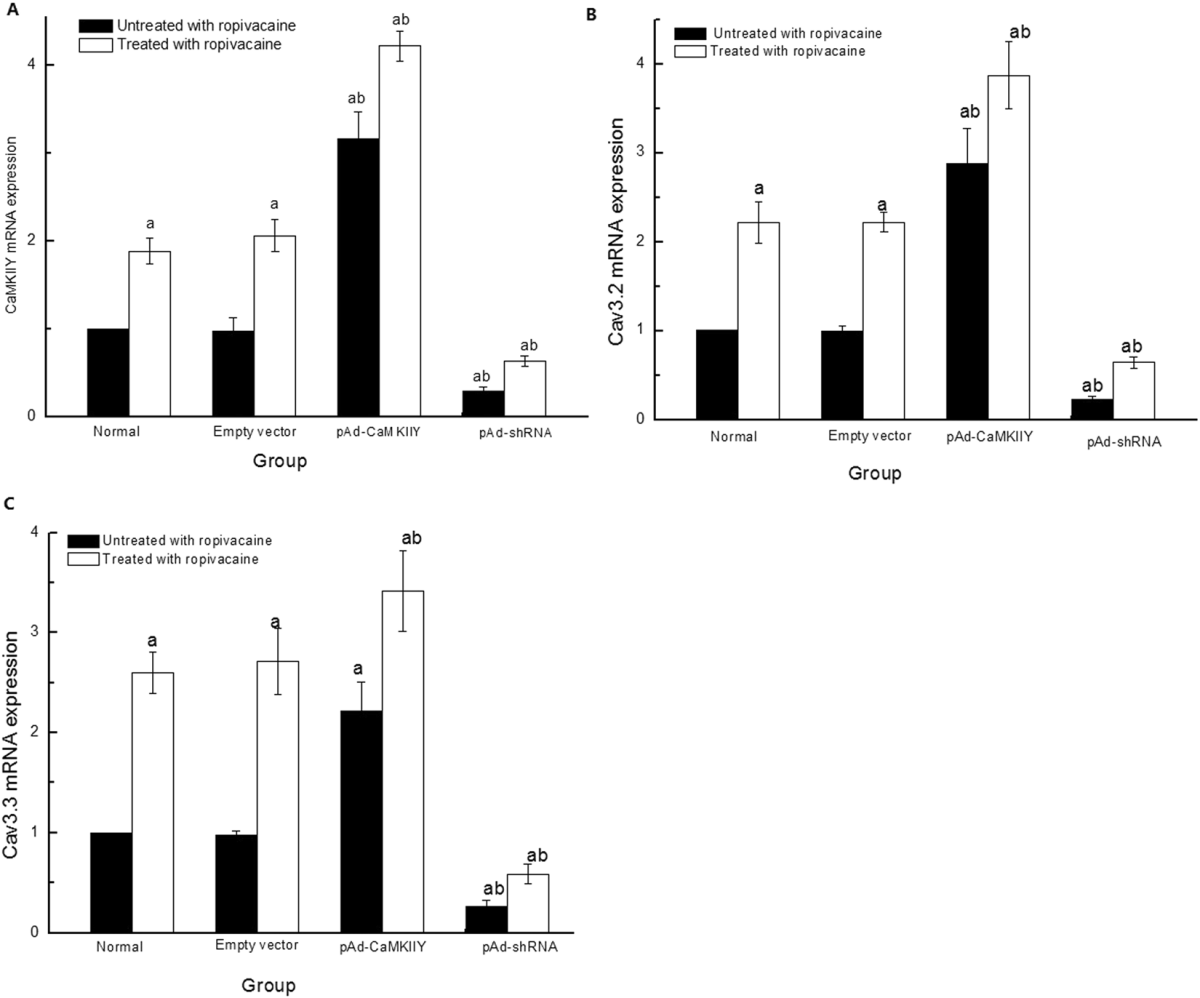
CaMKIIγ, Cav3.2 and Cav3.3 mRNA expression ($\overline{{\rm{x}}}$ ± s, n = 6). (**A**) CaMKIIγ mRNA expression; (**B**) Cav3.2 mRNA expression; (**C**) Cav3.3 mRNA expression. ^a^ *P* < 0.05 vs. Normal group; ^b^ *P* < 0.05 vs. Normal + R group.

CaMKIIγ mRNA expression was up-regulated in the pAd-CaMKIIγ group and down-regulated in the pAd-shRNA group compared to normal cells (Fig. 5A). There were no differences between cells in the Normal and Empty vector groups. These data suggest that the generated pAd-CaMKIIγ and pAd-shRNA adenovirus vectors successfully over-expressed and inhibited CaMKIIγ mRNA expression. CaMKIIγ mRNA expression in cells treated with ropivacaine hydrochloride also increased significantly compared to cells not treated with ropivacaine hydrochloride.

We measured Cav3.2 and Cav3.3 mRNA expression to examine the effects of CaMKIIγ. Cav3.2 mRNA and Cav3.3 mRNA expression in the pAd-CaMKIIγ group was up-regulated and down-regulated in the pAd-shRNA group (Fig. 5B and C). These data demonstrate that CaMKIIγ regulated Cav3.2 and Cav3.3 expression. Cav3.2 and Cav3.3 mRNA expression increased significantly in cells treated with ropivacaine hydrochloride compared to the untreated group.

### CaMKIIγ, p-CaMKIIγ, Cav3.2 and Cav3.3 protein expression

**Figure 6:**
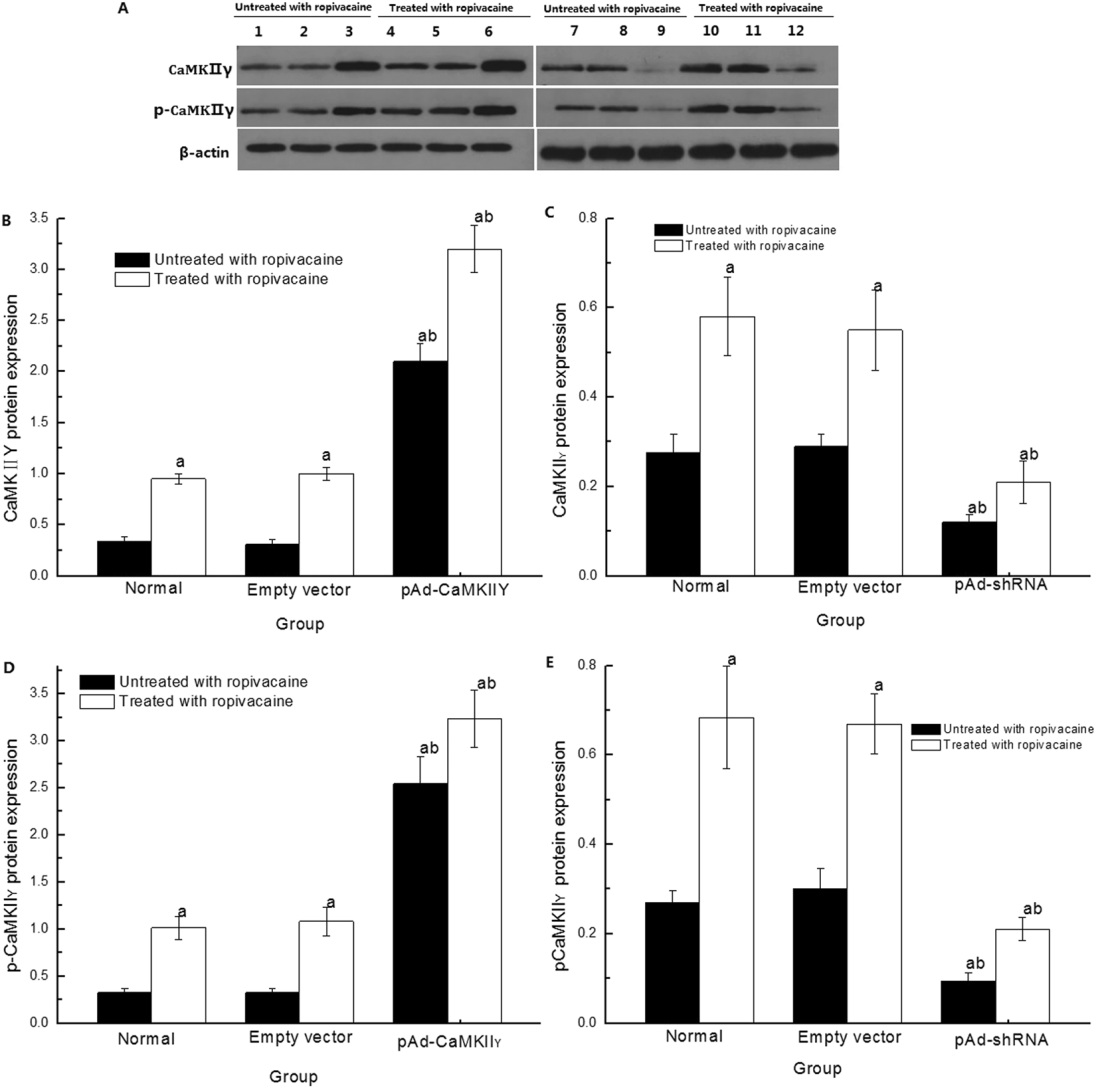
CaMKIIγ and p-CaMKIIγ protein expression detected with Western blotting. (**A**) Representative bands for CaMKIIγ and p-CaMKIIγ protein expression. Lanes 1 and7: Normal group; Lanes 2 and 8: Empty vector group; Lane 3: pAd-CaMKIIγ group; Lanes 4 and 10: Normal + R group; Lanes 5 and 11: Empty vector + R group; Lane 6: pAd-CaMKIIγ + R group; Lane 9: pAd-shRNA group; Lane 12: pAd-shRNA + R group. (**B**–**E**) CaMKIIγ and p-CaMKIIγ protein expression statistic data. ($\overline{{\rm{x}}}$ ± s, n = 6). ^a^ *P* < 0.05 vs. Normal group; ^b^ *P* < 0.05 vs. Normal + R group.

CaMKIIγ and p-CaMKIIγ protein expression in cells in the pAd-CaMKIIγ group significantly increased and decreased in the pAd-shRNA group compared to the Empty vector and Normal groups (Fig. 6). CaMKIIγ and p-CaMKIIγ protein expression of cells treated with ropivacaine hydrochloride group was obviously increased compared to cells in the untreated group. However, CaMKIIγ and p-CaMKIIγ protein expression increased in cells in the pAd- CaMKIIγ + R group and decreased in the pAd-shRNA + R group compared to the Normal + R and Empty vector + R groups.

**Figure 7:**
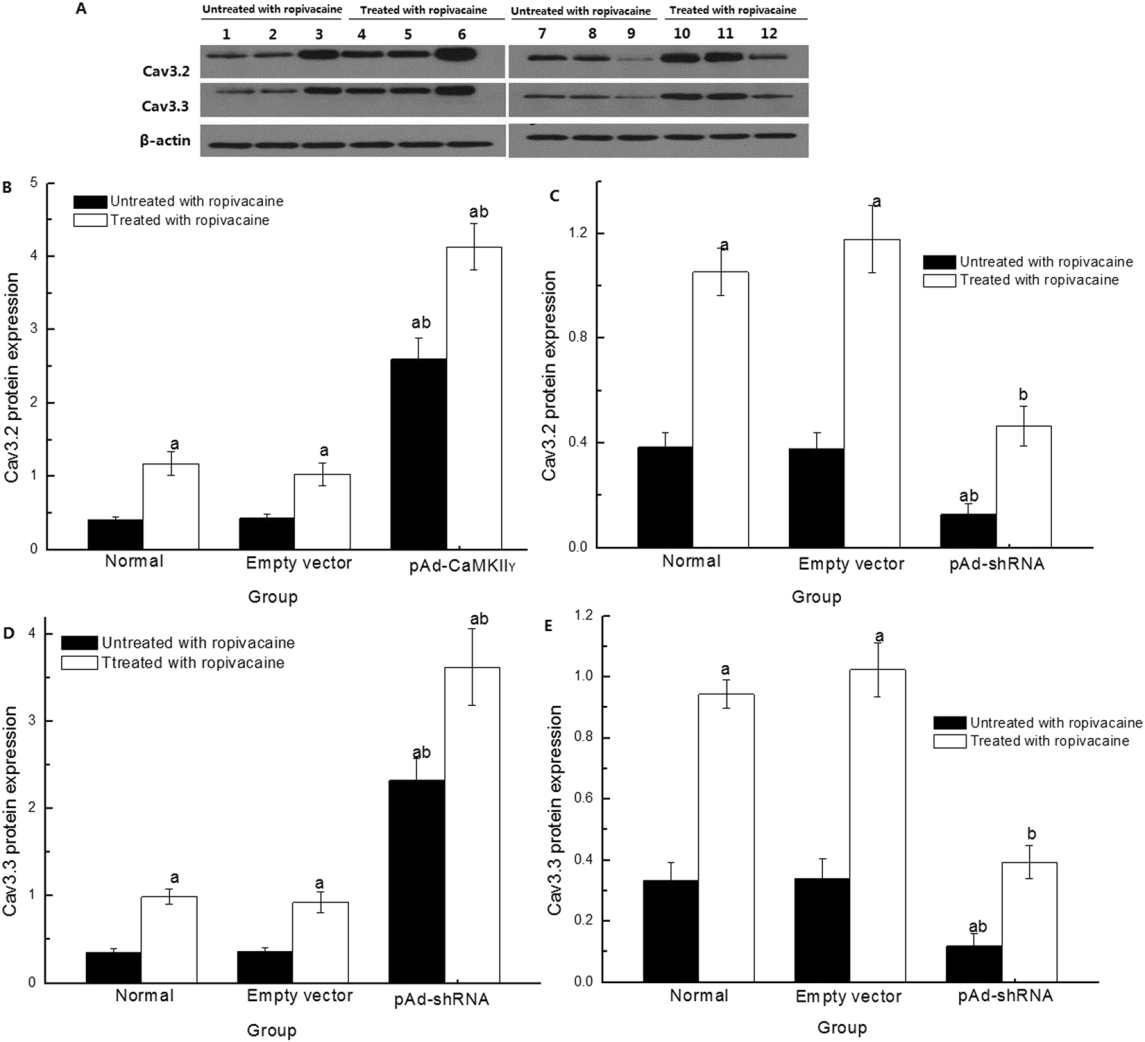
Cav3.2 and Cav3.3 protein expression detected with Western blotting. (**A**) Representative bands for Cav3.2 and Cav3.3 protein expression. Lanes 1 and 7: Normal group; Lanes 2 and 8: Empty vector group; Lane 3: pAd-CaMKIIγ group; Lanes 4 and 10: Normal + R group; Lanes 5 and 11: Empty vector + R group; Lane 6: pAd-CaMKIIγ + R group; Lane 9: pAd-shRNA group; Lane 12: pAd-shRNA + R group. (**B–E**) Cav3.2 and Cav3.3 protein expression ($\overline{{\rm{x}}}$ ± s, n = 6). ^a^ *P* < 0.05 vs. Normal group; ^b^ *P* < 0.05 vs. Normal + R group.

We also detected Cav3.2 and Cav3.3 protein expression.Cav3.2 and Cav3.3 protein expression was up-regulated in cells in the pAd-CaMKIIγ group and down-regulated in the pAd-shRNA group (Fig. 7). These data suggest that CaMKIIγ regulated Cav3.2 and Cav3.3 expression. Cav3.2 and Cav3.3 protein expression in cells treated with ropivacaine hydrochloride group increased significantly compared to the untreated group. However, the expression of Cav3.2 and p-Cav3.3 protein increased in cells in the pAd- CaMKIIγ + R group and decreased in the pAd-shRNA + R group compared to the Normal + R and Empty vector + R groups.

## Discussion

Local anesthetic neurotoxicity depends on concentration and exposure time^6, 26^. Higher concentrations and the longer exposure times generally induce more serious neurotoxicity. Therefore, local anesthetics are generally used at the lowest effective concentration to reduce neurotoxicity in clinical settings. Ropivacaine hydrochloride exhibits a lower toxicity than bupivacaine hydrochloride^27, 28^, but it also produces nerve damage in nerve block^7, 8^. DRG is the target of local anesthetics in spinal anesthesia, and DRG toxicity damage causes abnormal sensation. The present study investigated the effects of ropivacaine hydrochloride on DRG neurons. To determine the concentration and exposure time of ropivacaine hydrochloride, we initially examined normal DRG cell activity after treatment with different ropivacaine concentrations (1 mM, 2 mM, 3 mM, 4 mM, and 5 mM) and exposure times (0.5 h, 1 h, 2 h, 4 h, 6 h and 8 h). The results demonstrated that DRG cell viability decreased with increased ropivacaine concentrations and exposure time. DRG cell activity decreased to 50% at 3 mM of ropivacaine hydrochloride and a 4-h exposure time. Therefore, the parameters were used in subsequent experiments. This concentration of ropivacaine hydrochloride (3 mM) is approximately equal to 0.1 percent weight in volume, which is generally used in postoperative epidural and labor analgesia.

We generated pAd-CaMKIIγ and pAd-shRNA vectors to infect DRG and investigate the role of CaMKIIγ in ropivacaine hydrochloride-induced DRG neurotoxicity. Western blotting and qRT-PCR revealed that pAd-CaMKIIγ DRG cells up-regulated and pAd-shRNA DRG down-regulated CaMKIIγ expression. We examined the effect of ropivacaine hydrochloride on cell activity, apoptosis rate, and ultrastructure in ropivacaine hydrochloride-treated and untreated DRG cells. The results demonstrated that ropivacaine hydrochloride treatment caused injury, a decrease in viability and increase in apoptosis rate in all treated groups. However, damage in the DRG was more serious in the pAd- CaMKIIγ + R group and less serious in the pAd-shRNA + R group than the Normal + R and Empty vector + R groups. These data suggest a role of CaMKIIγ in ropivacaine hydrochloride neurotoxicity.

CaMKII is an important factor in the regulation of synaptic transmission and neuronal function^29–31^. Our results demonstrated that the up- or down-regulation of CaMKIIγ expression in DRG cells altered the expression of Cav3.2 and Cav3.3 T-type calcium channels. A previous study found that activation of CaMK II directly stimulated Cav3.2-type T calcium channels and increased Cav3.2 T calcium currents. A CaMK II inhibitor significantly reduced Cav3.2 T calcium currents *in vitro* ^25^. Ser1198 of the Cav3.2 type calcium channel is the phosphorylation site of CaMK II activation, which is the molecular basis of CaMK II regulation of Cav3.2-type calcium channels. CaMK II activation mediated the phosphorylation of Ser1198 of the Cav3.2 type calcium channel and up-regulated Cav3.2 expression. Our previous studies demonstrated that the neurotoxicity of local anesthetics involved T-type calcium channels^22, 23^. The inhibition of T-type calcium channel expression using specific calcium channel inhibitors or RNA interference reduced the neurotoxicity of local anesthetics, and the up-regulation T-type calcium channel expression aggravated local anesthetic neurotoxicity.

## Conclusion

In conclusion, CaMKIIγ may play a role in ropivacaine hydrochloride-induced DRG neurotoxicity. CaMKIIγ regulation of Cav3.2 and Cav3.3 T-type calcium channels participated in ropivacaine hydrochloride neurotoxicity. This study has one primary limitation. We observed the effects of ropivacaine hydrochloride on primary DRG cells *in vitro*, and the experimental results may be different in animal model experiments *in vivo*. The results of our *in vitro* experiments should be validated in animal models.

## Materials and Methods

### Isolation and culture of rat dorsal root ganglion cells

All animal procedures were performed in accordance with national and international animal care and ethical guidelines, and the Institutional Animal Care and Usage Committee at the First People’s Hospital of Foshan City, Guangdong province, China approved all procedures. Neonatal Sprague-Dawley rats were purchased from the Experimental Animal Center of Guangdong province, China. Rats were anesthetized with sevoflurane and euthanized via decapitation. The area of incision was disinfected using iodophor, and the skin and muscle of the back were cut to expose the spine. The spinal cord and DRG were isolated under a dissecting microscope, and DRG were placed in a sterilized pre-cooled phosphate-buffered saline (PBS) solution. Ganglion membranes were removed, and the DRG were transfer to a 15-ml centrifuge tube for centrifugation at 1000 rpm for 2 min. The supernatant was discarded, and DRG were rinsed twice in PBS. Four milliliters of 0.125% trypsin was added to the ganglia, which were incubated at 37 °C for 20 min. DMEM complete culture medium (4 ml) was added to the ganglia to terminate the digestion, and the tubes were centrifuged at 1000 rpm for 2 min. Ganglia were washed twice in PBS, and 2 ml of Neurobasal medium (4.5 g/L containing D- glucose, 2 mmol/L L-, 1% FBS 20 ml/L B-27, glutamine additive, 10 μg/ml NGF penicillin 100 U/ml, streptomycin, 100 μg/ml) was added to suspend the DRG neurons. DRG neurons were filtered through a 400-mesh stainless steel filter and seeded in cell culture plates at a density of 2~3 × 10^5^/ml for incubation at 37 °C and 5% CO_2_ for 48 h. New culture media supplemented with cytosine arabinoside (5 mM final concentration) was exchanged after 48 h to inhibit non-neuronal cell proliferation. Cells were incubated at 37 °C in 5% CO_2_ for 96 h. Normal culture media without cytosine arabinoside was exchanged every 3 days.

### Generation of pAd-shRNA and pAd-CaMK II γ vector

We generated a pAd-shRNA vector to down-regulate CaMK II γ mRNA expression in DRG cells. Briefly^32^, we designed the shRNA primer according to the rat Genebank CaMK II gene (NM_133605.1) 824-842: 5′-TTTGGGTCAACAGTGGCATCCATTTCAAGACGATGGATGCCACTGTTGACCTTTTTTG-3′; 5′-AGCTCAAAAAAGGTCAACAGTGGCATCCATCGTCTTGAAATGGATGCCACTGTTGACC-3′ (synthesized by Jinsirui Ltd., Nanjing, China). The primer annealed to form a double strand, which was connected to a pYr-1.1 vector (Wuhan Biobuffer Biotech Service Co. LTD., China) to generate the pYr-1.1-shRNA plasmid. The pYr-1.1-shRNA was recombined with the pAd/PL-DEST adenovirus vector (Invitrogen Technology Co., LTD, USA) to generate pAd-shRNA, which was transfected into HEK 293 cells for virus amplification and infection of DRG neurons.

We generated the pAd-CMK II vector to up-regulate the CaMK II γ mRNA expression in DRG cells. We synthesized the full-length CaMK II γ (NM_133605.1) cDNA, which was connected to the pYr-adshuttle-4 vector (Wuhan Biobuffer Biotech Service Co. LTD, China) to generate the pYr- CaMK IIγ plasmid. The pYr- CaMK IIγ plasmid was recombined with pAd/PL-DEST adenovirus vector to generate the pAd- CaMK IIγ adenovirus vector. The pAd- CaMK IIγ adenovirus vector was transfected into HEK 293 cells for virus amplification and infection of DRG neurons. Real-time PCR and Western blotting were used to identify CaMK II γ expression in pAd-shRNA-DRG and pAd-CaMK IIγ-DRG neurons.

### Experimental protocol

We initially examined the effects of different ropivacaine hydrochloride concentrations and exposure times (AstraZeneca Pharmaceutical Co. Ltd., UK) on dorsal root ganglion neurons. Dorsal root ganglion neurons were treated with ropivacaine hydrochloride (final concentrations: 1, 2, 3, 4, and 5 mM) for 0.5 h, 1 h, 2 h, 4 h, 6 h and 8 h, and cell viability was measured to investigate neurotoxicity. DRG cells not treated with ropivacaine hydrochloride (“Untreated group”) were divided into four groups: normal DRG (Normal group), Empty vector in DRG (Empty vector group), pAd-CaMKIIγ DRG (pAd-CaMKIIγ group), and pAd-shRNA DRG (pAd-shRNA group). DRG cells treated with ropivacaine hydrochloride were divided into 4 groups: Normal + R group, Empty vector + R, pAd-CaMKIIγ + R group and pAd-shRNA + R group. Cells treated with ropivacaine hydrochloride group were incubated with 3 mM ropivacaine hydrochloride for 4 h.

### Cell viability detection using MTT

Cells in each group were seeded in 96-well plates at a density of 1 × 10^5^ /ml and incubated at 37 °C in 5% CO_2_. Cells that were untreated or treated with ropivacaine hydrochloride (3 mM) received new normal culture media after 4 h. A small volume (20 μl) of 5 mg/ml 3-(4,5-dimethyl-2- thiazolyl)-2,5-diphenyl-2-tetrazolium bromide (MTT, Beyotime biotech Co. Ltd., China) was added to each well and incubated at 37 °C in 5% CO_2_ for 4 h. The culture media were discarded, and 150 μl DMSO was added to each well to dissolve purple crystals. Optical density was detected at 570 nm and 630-nm absorbance wavelengths (OD_570_ and OD_630_), and the difference between the two wavelengths was calculated. DRG cells in the normal group were set as 100%, and the other groups were normalized to normal group values.

### Apoptosis rate

Cells were seeded into 24-well plates at a density of 2 × 10^5^ /ml, with 500 μl per well. Cells were treated or untreated with 3 mM ropivacaine hydrochloride for 4 h and collected to detect the apoptosis rate using an Annexin V apoptosis detection kit-APC (Affymetrix Inc., San Diego, CA, USA). Briefly, cells were collected via centrifugation at 2000 rpm for 3 min, and the supernatants were discarded. Cells were suspended in 1× binding buffer. These steps were repeated twice, and 100 μl of an annexin V antibody (1:20) was added to the cells. Cells were incubated in a dark box at room temperature for 15 min. Cells were washed with 1× binding buffer, and 10 μl 2% 7-A-A-D was added to the cells for a 5-min incubation in dark box. Cells were further diluted with 190 μl 1× binding buffer, and the apoptosis rate was detected using flow cytometry.

### Ultrastructure of DRG cells

Ropivacaine hydrochloride-treated (3 mM) or untreated cells in each group were collected after a 4-hr incubation and centrifuged at 1000 rpm for 10 min. The supernatants were discarded, and cells were fixed with 2.5% glutaraldehyde. Cells were washed three times with cold PBS buffer, fixed with 1% osmium tetroxide for 15 min at 4 °C, and washed three times with cold PBS. Dehydration was performed at room temperature in a series of acetone gradients: 50% acetone solution for 10 minutes, 70% acetone solution for 10 minutes, 90% acetone solution for 20 minutes and 100% acetone solution for 30 minutes. Cells were incubated overnight in 1 ml of an embedding agent at room temperature. Cells were transferred to a capsule module, which was filled with embedding agent. The capsule module was placed in an oven for solidification at 60 °C for 2 hours. The solidified block was sliced into 1-μm slices, which were stained with hematoxylin and eosin. Cell images were observed under a microscope, and the location of ultrathin sections was determined and labeled. A triangular glass knife was installed, and the paraffin blocks were fixed and cut into 50~70 nm ultrathin sections. Sections were stained with sodium acetate and lead citrate and washed. The ultrastructure of DRG cells was observed under a transmission electron microscope (HITACHI transmission electron microscope HT7700, Japan).

### qRT-PCR

Total RNA was extracted from cells after treatment or non-treatment with 3 mM ropivacaine hydrochloride. Briefly, cells were lysed on ice in 200 μl TRIzol. A five-fold volume of chloroform was added to the lysed cells and mixed for 5 min. Lysed cells were centifuged for 15 min at 14,000 rpm and 4 °C. The upper layer of RNA was collected, and the same volume of isopropanol was added. Tubes were centrifuged for 10 min at 14,000 rpm and 4  °C. The supernatant was discarded, and the pellet was washed with 70% ethanol. Tubes were centrifuged for 10 min at 1000 rpm and 4 °C, and the supernatant was discarded. Pellets were air-dried for 15 min. Nuclear enzyme-free water (30 μl) was added to dissolve the total RNA, and the RNA concentration measured. RNA (5 μg), 2 mM dNTP (5 μl), and 10 pM random primers (1 μl) were mixed, and nuclear enzyme-free water was added to 37 μl. Solutions were incubated at 65 °C for 5 min. First-stand buffer (10 μl, 5 × ), 0.1 M DTT (2 μl), and mLv reverse transcriptase (1 μl) were added to the mixture and incubated at 42 °C for 1 h and 70 °C for 15 min. The cDNA product (5 μl) was mixed with 10 pM CaMK II γ, Cav3.2, and Cav3.3 mRNA primers (0.5 μl) (synthesized by Jinsirui Ltd., Nanjing, China, Table 1), and 6 μl Sybergreen (Bioscience Inc., USA) was added to the PCR reaction. The following reaction parameters were used: set 95 °C 10 min, one repeat, 95 °C 15 s, 60 °C 30 s, 95 °C 15 s, 40 repetitions. Fluorescence signals were measured, and the Ct value. 2^−ΔCt^ (Ct cycle threshold), was set as the quantity of gene expression. ΔCt = [Ct (target gene) - Ct (beta -actin)]. The normal group DRG mRNA expression was set as 1, and the other groups were normalized to the normal group.

**Table 1:** CaMKIIγ, Cav3.2, Cav3.3 mRNA primer sequences.

Gene	Primer	Product size
β-actin	F: 5′-CACGATGGAGGGGCCGGACTCATC -3′ R: 5′-TAAAGACCTCTATGCCAACACAGT-3′	240 bp
Cav3.3	F: 5′-GACCAGCAGCCAGTGACGAA-3′ R: 5′-CACGACCACGCCCACAAACA-3′	108 bp
Cav3.2	F: 5′-GGAGTTTGATGATGACATAGAGG-3′ R: 5′-GGAAGATGAAGACAAGGACCAC-3′	197 bp
CaMKIIγ	F: 5′-TTGCTGCTGGCGAGTAAATG-3′ R: 5′-TAGGGATCTTTCCTCAAGACCTCA-3′	149 bp

### Western blotting

Cells were treated or not treated with 3 mM ropivacaine hydrochloride, and 200 μl of a pre-cooled lysis solution was added to the collected cells for 30 min. Ultrasonic waves for 30 s crushed the cells, and cells were centrifuged at 4 °C and 12,000 rpm for 20 min. Total protein was incubated in 95 °C water for 5 min. Protein (20 μg) from each group was subjected to SDS electrophoresis at 60 V in a concentrating gel electrophoresis for 30 min and 100 V separation gel electrophoresis for 70 min. The target strip was cut based on the position of the protein marker and transferred to a PVDF membrane. The film was soaked in TBST and incubated in a close solution at room temperature on a shaking bed for 1 h. The film was washed twice with TBST and incubated with antibodies (1:200) against CaMKII γ, pCaMKII γ, Cav3.2, Cav3.3 and β-actin (Sigma Company, USA) at 4 °C overnight. The film was washed with TBST 3 times and incubated in a second antibody (1:1000) for 1 h. Films were washed 3 times with TBST, and the chemiluminescence reagent was added for 1 min. The film was quickly wrapped and placed in a cassette with Kodak X- film for exposure. Quantity One image analysis software was used to analyze the absorbance value of the target band using the absorbance value of β-actin band as the reference. The ratio indicated target protein expression level.

### Statistical analysis

Data are expressed as Means ± SD. A factorial design was used for statistical analyses. One-way analysis of variance (one-way ANOVA) was used for comparisons between groups. The LSD method was used for multiple comparisons.
